# Differences in Psychological Health and Weight Loss after Bariatric Metabolic Surgery between Patients with and without Pain Syndromes

**DOI:** 10.1007/s11695-024-07171-y

**Published:** 2024-03-18

**Authors:** Johanna E. Pyykkö, Max Zwartjes, Max Nieuwdorp, Nienke van Olst, Sjoerd C. Bruin, Arnold W. van de Laar, Robbert Sanderman, Mariët Hagedoorn, Victor E. A. Gerdes

**Affiliations:** 1 Department of Health Psychology, Faculty of Medical Sciences, University of Groningen, University Medical Center Groningen, Antonius Deusinglaan 1, 9713 AV Groningen, Netherlands; 2 Department of Internal Medicine, Spaarne Gasthuis, Spaarnepoort 1, 2134 TM Hoofddorp, Netherlands; 3 Department of Vascular Medicine, Amsterdam UMC, Meibergdreef 9, 1105 AZ Amsterdam, Netherlands; 4 Department of Metabolic and Bariatric Surgery, Spaarne Gasthuis, Spaarnepoort 1, 2134 TM Hoofddorp, Netherlands

**Keywords:** Pain syndromes, Bariatric metabolic surgery, Psychological wellbeing

## Abstract

**Purpose:**

Chronic pain and obesity often co-occur, negatively affecting one another and psychological wellbeing. Pain and psychological wellbeing improve after bariatric metabolic surgery (BMS), however, it is unknown whether psychological wellbeing improves differently after weight loss between patients with and without chronic pain. We investigated whether weight loss is associated with greater psychological wellbeing and functioning change after BMS, comparing patients with and without preoperative pain syndromes.

**Methods:**

Depression, health-related quality of life, self-esteem, self-efficacy to exercise and controlling eating behaviours, physical activity, and food cravings were measured before and 24 months after BMS among 276 patients with obesity. The presence of preoperative chronic pain syndromes was examined as a moderator for the relationship between 24-month weight loss and changes in psychological outcomes.

**Results:**

Chronic pain syndromes were present among 46% of patients. Weight loss was associated with greater improvement in health-related quality of life, self-efficacy to exercise and controlling eating behaviours, self-esteem and greater amelioration in food cravings. Pain syndromes only moderated negatively the relationship between the postoperative weight loss and change in self-efficacy to control eating behaviours (*b* = -0.49, CI [-0.88,-0.12]).

**Conclusion:**

Patients with and without chronic pain showed similar improvements in weight and psychological wellbeing and behaviours after BMS. The relationship between weight loss and the improvement of self-efficacy to control eating behaviours was weaker among patients with chronic pain syndrome. Further work, measuring pain severity over time, is needed to shed light on the mechanism underlying pain and postoperative change in psychological wellbeing and weight loss.

**Graphical Abstract:**

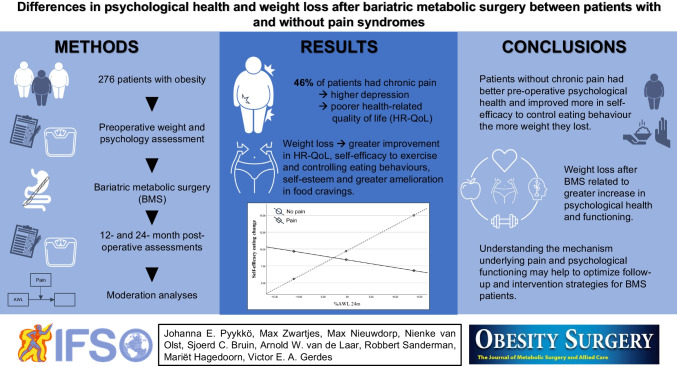

## Introduction

Increasing research evidence suggests that obesity and chronic pain are significantly related to each other [1, 2]. Globally, more than 1.9 billion adults have overweight (Body mass index (BMI) > 25 kg/m^2^) and over 650 million individuals have or live with obesity (BMI > 30 kg/m^2^) [3]. Up to 40% of adults with obesity experience chronic pain [4, 5], whereas 10% of individuals with healthy weight report chronic pain worldwide [6]. Chronic pain is characterized as recurrent or persisting pain that extends a period of 3 months or longer [7], and chronic pain conditions most related to obesity include musculoskeletal and joint pain [8, 9], fibromyalgia [10], chronic headache or migraine [11], abdominal pain or irritable bowel syndrome (IBS) [12], and lower back pain among others [2, 13]. Under the recent classification system by the task force of the International Association for the Study of Pain (IASP), the chronic pain syndromes in obesity mostly fall under the categories of chronic musculoskeletal pain (symptomatic osteoarthritis of knee, lower back, hip, or other chronic musculoskeletal pain syndromes) as well as chronic primary pain (fibromyalgia, nonspecific back pain, and IBS) [7]. Pain becomes more frequent, persistent and intense as weight increases [5, 14–16]. Furthermore, chronic pain limits physical activity, and self-efficacy to exercise and has been related with depressive symptoms and poorer quality of life among individuals with overweight and obesity [14, 17–21]. Adults with obesity reported to overeat to soothe their pain, which led to feelings of guilt and triggered further unhealthy eating [19, 22].

Given that various mechanical, physiological, psychological and behavioural mechanisms have been proposed to link obesity and pain in a reciprocal manner, it is likely that weight loss impacts these pathways [1, 23]. Indeed, accumulating evidence shows that weight loss can alleviate pain related to obesity, improve quality of life and reduce the effect of pain on disability [2]. Bariatric metabolic surgery leads to significant weight loss, amelioration or complete resolution of medical problems associated with obesity and significant improvement in health-related quality of life and depression [24–26]. Also pain experiences and syndromes ameliorate significantly as weight decreases after the surgery [27–29]. However, no previous study has investigated whether patients with obesity and a pain syndrome have similar response to bariatric metabolic surgery in terms of psychological wellbeing and functioning as those without a pain syndrome. A previous study found no differences in early postoperative (1- and 3 -month postoperative) psychological or medical complications between patients with and without pain [30]. Given that pain and obesity influence each other reciprocally, and that pain amelioration is associated with better psychological wellbeing, we hypothesized patients with a pain syndrome to experience greater improvements in their psychological wellbeing after bariatric metabolic surgery than patients without pain with the same weight loss results. Using recent data from the BARIA and DIABAR studies [31, 32], we set out to evaluate whether weight loss leads to more pronounced improvements in depressive symptoms, health-related quality of life, self-esteem, self-efficacy to exercise, physical activity, self-efficacy to control eating behaviours and food cravings among patients with a pain syndrome compared to those without a pain syndrome.

## Methods

### Study Design and Participants

This study involves data from two ongoing prospective research projects, namely the BARIA- and DIABAR- studies [31, 32]. Both studies were approved by the by the Medical Research Ethics Committee of the Academic Medical Center Amsterdam (approval code NL55755.018.15 and NL61882.048.17), and conducted in accordance with the Declaration of Helsinki and the Medical Research Involving Human Subjects Act (WMO).

Participants were included in the study if they were over 18 years of age, had a BMI over 35 kg/m^2^ and medical conditions associated with obesity or a BMI above 40 kg/m^2^, had attempted weight loss under supervision, and provided written informed consent. A research physician assessed participants before the operation, and 12, and 24 months after the operation. For this study, we used data collected until 27 September 2022 and included only patients with 24-month follow-up data. Participants’ socio-demographic details and psychological profile were measured during the preoperative visit with a self-report survey including the NEO-FFI questionnaire [33] to assess neuroticism and conscientiousness, the Rosenberg self-esteem scale [34], and The Modified and Brief Experiences in Close Relationships scale [35] to measure attachment style. The included scales were Dutch versions of validated psychological instruments. The presence of a chronic pain syndrome (IBS, fibromyalgia, chronic headache or migraine, chronic lower back pain (CLBP), chronic musculoskeletal pain (CMP) from structural osteoarticular changes, use, number and dose of analgesics, and use of antidepressants) was obtained from the patients’ electronic medical records. Patients were diagnosed to have a chronic pain condition if they met the IASP criteria of chronic pain described above. Participants were grouped based on the presence of at least one preoperative pain condition into either having a pain syndrome or not. Participants filled the self-report survey again at the follow-up visits.

### Measures

#### Weight and Weight Loss

A clinician measured the participants’ weight during hospital visits. Weight loss was measured as change in BMI, percentage of adjustable weight loss ($\%{\text{AWL}}=\left(\left({BMI}_{pre-op}-{BMI}_{post-op}\right)/{BMI}_{pre-op}-13\right)\times 100$ [36] and percentage of total weight loss (%TWL).

#### Depression

The Center for Epidemiology Studies Depression Scale Revised version [37] is a 20-item questionnaire used to measure depression. The total score ranged from 0 to 60 with higher score indicating more severe depressive symptoms.

#### Health-Related Quality of Life

The Impact of Weight on Quality of Life [38, 39] questionnaire includes 31 items assessing health-related quality of life among populations with overweight. The total score ranged from 0 to 100 with higher score denoting a better quality of life.

#### Self-Efficacy Exercise

One’s belief in their capability to perform regular physical exercise was assessed with the 10- item Exercise Self-Efficacy Scale (ESES) [40, 41].

#### Physical Activity

Time spent on various forms of exercise (i.e., cycling, aerobic exercise) in the past week was collected using The Exercise Behaviour scale [42]. The six items of the scale were summed into a total score indicating the amount of time patients spent exercising, ranging from 0 to 720 min/week.

#### Self-Efficacy Eating

The Weight Efficacy Lifestyle Questionnaire [43]) was used to assess participants’ self-efficacy for controlling their eating behaviour in specific situations on a scale from 0 to 100.

#### Food Craving

The desire to eat in different situations was assessed with the G-Food Craving Questionnaire-Trait (FCQ-T) [44]. The scale consists of 21 items and ranges from 0 to 100, with higher scores denoting more cravings for food.

### Statistical Analysis

Data management and analysis were performed using SPSS 27.0 (2022) [45]. The demographic characteristics and presence of pain conditions of the sample were summarized using descriptive statistics and presented in Table 1. Independent-sample *t-* tests for normally distributed variables and Wilcoxon Rank Sum Test for paranormally distributed variables were calculated to examine differences in the preoperative mean psychological characteristics and wellbeing scores, and weight between participants in the pain syndrome versus no pain syndrome group. Changes in psychological wellbeing and functioning variables were calculated for each study variable (i.e., depression, health-related quality of life, self-esteem, self-efficacy, physical activity, and food cravings) by deducting the 24-month postoperative score from the preoperative score, and used as outcome variables. Correlational analyses were performed to examine the associations between the study variables. We conducted multiple moderation tests using the SPSS macro PROCESS [46], with 24-month postoperative weight loss as the antecedent variable, changes in psychological wellbeing or functioning as the dependant variables, and presence of pain syndromes as a moderator. The antecedent variable was mean-centred, and the presence of pain syndrome was included as a dummy coded moderator with 0 indicating no pain and 1 indicating presence of pain. We estimated the unstandardized regression coefficients, standard errors, *t*- and *p*-values and the 95%-Bias-corrected Bootstrap confidence intervals with PROCESS. Significant interactions were probed with the percentile approach.

**Table 1:** Sociodemographic Characteristics of Participants (*n* = 276)

	*n*	*%*
Gender, female	210	76.1%
Age (mean, SD) [range 20.4–65.3 years]	47.85	10.38
Race		
Caucasian	246	89.1%
South American	10	3.6%
Mediterranean	5	1.8%
Asian	6	2.1%
Other	9	3.2%
Marital status		
Married/ partnered	206	74.6%
Single	49	17.8%
Divorced/widowed	21	7.6%
Relationship duration (years)	14.35	13.5 8
Having children, yes (median = 2 children)	211	76.4%
Highest educational level		
Lower general education / primary education, or a part of it	4	1.4%
General education/ high school	86	31.2%
Secondary vocational education	116	42.0%
Higher professional education	48	17.4%
Scientific education	16	5.8%
Other	6	2.2%
Employment		
Employed (full or part time)	218	79.0%
Disabled for work	17	6.2%
Homework, voluntary or unpaid work	26	9.4%
Searching for work	9	3.3%
Retired	3	1.1%
Study	3	1.1%
Pain conditions: unique cases		
Irritable bowel syndrome (IBS)	7	2.5%
Fibromyalgia	5	1.8%
Migraine	13	4.7%
Chronic lower back pain (CLBP)	22	8.0%
Chronic musculoskeletal pain from structural osteoarticular changes (CMP)	13	4.7%
Analgesics use	18	6.5%
Pain conditions: overlap with other conditions allowed:		
IBS	19	6.9%
Fibromyalgia	24	8.7%
Migraine	25	9.1%
CLBP	66	23.9%
CMP	41	14.9%
Analgesics use	74	26.8%
Antidepressant use	45	16.3%
Combinations		
Chronic pain	127	46.0%
Chronic pain & analgesics use	145	52.5%

*SD* standard deviation

Chronic pain = one or more of the following conditions: fibromyalgia, migraine, IBS,CLBP,CMP

## Results

### Participant Characteristics

Of the 589 initially included patients, preoperative data was available for 560 individuals, with missing data attributed to non-completion of the psychological survey, study withdrawal, death, and patients waiting for surgery. An additional 45 patients had not undergone surgery, mainly due to study withdrawal, insufficient weight loss, and pending surgery. Surgical data was missing from 12 patients. For the 12-month postoperative period, data was available for 360 patients, with missing data linked to various factors such as study withdrawal, pregnancy, death, non-attendance, failure to complete the survey, and a follow-up period shorter than 12 months. In the 24-month postoperative assessment, data was absent for 84 patients, primarily due to study withdrawal, pregnancy, non-attendance, failure to complete the psychology questionnaire, and a follow-up period shorter than 24 months. Consequently, data for the 24-month assessment was available for 276 patients, of whom 210 (76.1%) were female, with a mean age of 47.9 (SD = 10.4) years. Laparoscopic Roux-en-Y Gastric Bypass was performed on 248 (89.9%) patients. One or more of the chronic pain conditions was present among 127 (46.0%) patients. The most common condition was CLBP (23.9%), followed by CMP (14.9%), migraine (9.1%), fibromyalgia (8.7%), and IBS (6.9%). Furthermore, nearly 30% of all patients reported using analgesics and 16.3% used antidepressants. A more detailed overview of the demographic characteristics and of pain conditions is presented in Table 1. Patients with pain syndromes had significantly poorer preoperative health-related quality of life (Δ = -4.22, *p* = 0.04) and higher depressive symptoms (Δ = 1.95, *p* = 0.03) and BMI (Δ = 0.96, *p* = 0.03) than patients without pain. The patients did not differ on preoperative personality characteristics (neuroticism, conscientiousness, self-esteem or attachment style), self-efficacy to exercise or control eating behaviours nor on physical activity level or food cravings (Table 2). Weight loss 24 months after surgery was similar among patients with or without pain syndromes (%TWL = 27.89 (SD = 8.20) versus 28.98 (SD = 7.74), *p* = 0.26, Table 3).

**Table 2:** Differences in preoperative characteristics between patients with and without chronic pain

	Chronic pain					
	Yes (*n* = 127)		No (*n* = 149)			
	Mean	SD	Mean	SD	*p*	r
BMI	38.42	3.65	39.38	3.57	0.028	-0.14
Well being						
Depression*	9.72	8.02	7.77	6.07	0.026	0.10
Quality of life*	59.82	17.90	64.04	16.33	0.042	-0.12
Self-efficacy						
SE exercise*	31.39	5.88	32.70	5.34	0.367	-0.11
SE eating*	65.21	18.69	65.60	18.56	0.055	0.00
Health behaviour						
Physical activity*	152.72	130.47	166.51	132.70	0.387	-0.06
Food craving*	47.66	19.72	45.78	20.24	0.861	0.05
Personality						0.00
Neuroticism	29.88	8.80	28.72	8.41	0.440	0.06
Conscientiousness	46.92	6.42	47.35	6.78	0.267	-0.05
Self-esteem	19.98	5.40	20.55	5.08	0.593	-0.04
AS avoidance	21.59	9.37	20.09	7.98	0.151	0.06
AS anxiety	23.51	11.00	22.91	10.71	0.644	0.02

*N* for neuroticism and conscientiousness = 273, for self-esteem *n* = 271

*Wilcoxon rank sum test

**Table 3:** Results of t-tests for difference in postoperative weight loss

	Chronic pain				
	Yes		No		
	Mean	SD	Mean	SD	*p*
WL 12-m (BMI)	-11.39	2.98	-11.36	2.96	0.936
%TWL 12-m	29.47	6.69	28.88	7.04	0.473
%AWL 12-m	44.77	9.97	43.38	10.71	0.271
WL 24-m (BMI)	-11.20	3.38	-10.95	3.35	0.539
%TWL 24-m	28.98	7.74	27.89	8.20	0.259
%AWL 24-m	43.97	11.58	41.93	12.48	0.164

'No' group *n* at 24 months follow up is 148

### Moderation between Weight Loss and Psychological Outcomes

The descriptive statistics and correlations of the study variables are presented in Table 4. Correlation analysis showed significant relations between the 24-month postoperative %AWL, improvement in health-related quality of life, self-esteem, self-efficacy to exercise, and decrease in food cravings. Subsequently, presence of pain syndromes was examined as a moderator of the relation between 24-month postoperative weight loss and change in psychological wellbeing. Most moderation analyses yielded insignificant results, which are summarized in Table 5.

**Table 4:** Correlations between change scores in study variables and descriptive statistics

		1	**2**	3	4	5	6	7	8	9	10	11	12
1	Depression change	–											
2	HR-Quality of life change	-.22**	–										
3	Self-esteem change	-.39**	.39**	–									
4	Self-efficacy exercise change	-.20**	.23**	.31**	–								
5	Physical activity change	-.06	.14*	.10	.29**	–							
6	Self-efficacy eating change	-.25**	.20**	.24**	.19**	.13*	–						
7	Food cravings change	.11	-.33**	-.24**	-.11	.02	-.52**	–					
8	% total weight loss at 24 m	.04	.22**	.19**	.16*	-.04	.10	-.17**	–				
9	% adjustable weight loss at 24 m	.05	.22**	.18**	.15*	-.05	.10	-.18**	.99**	–			
10	Age	-.05	-.03	.04	-.04	.18**	.24**	-.09	-.21**	-.19**	–		
11	Gender	.05	.11	.09	.07	-.07	-.17**	.04	.24**	.24**	-.21**	–	
12	Chronic pain	-.00	.03	-.01	-.06	.03	-.01	-.08	.07	.08	.18**	.16**	–
	Min	-23.00	-83.61	-14.00	-24.00	-450.00	-60.88	-67.86	2.83	4.50	20.39	0.00	0.00
	Max	41.00	70.49	16.00	22.00	525.00	68.31	43.64	46.33	69.40	65.32	1.00	1.00
	Mean	0.49	16.60	2.29	2.45	42.99	8.90	-11.95	28.40	42.87	47.85	0.76	0.46
	SD	8.66	20.36	4.38	6.14	169.38	19.12	19.53	8.00	12.10	10.38	0.43	0.50
	Skewness	1.63	-0.43	-0.06	-0.46	0.13	0.08	-0.19	-0.19	-0.19	-0.64	-1.23	0.16
	Kurtosis	5.99	2.31	1.39	2.32	0.72	1.25	0.09	-0.15	-0.12	-0.19	-0.49	-1.99

** *p* < .01, * *p* < .05

*N* = 276, except for self-esteem *n* = 270, food craving *n* = 273, weight loss at 24 m *n* = 275

Pearson correlation coefficient is reported for all variables, except for Gender and Chronic Pain, the Spearman Rank order correlation is used

**Table 5:** Various regression models estimating the change in psychological outcomes

		Coeff	*SE*	*t*	*p*	LLCI	ULCI
Model 1. Depression change							
*R*^*2*^ = 0.016, *MSE* = 74.85, *F*(3, 271) = 1.06, *p* = .367							
Constant	*i*_*y*_	0.45	0.68	0.64	.524	-0.78	1.89
AWL 24 m (X)	*b*_*1*_	-0.02	0.06	-0.34	.736	-0.12	0.12
Chronic pain (W)	*b*_*2*_	0.00	1.04	0.00	1.000	-2.02	2.05
Interaction	*b*_*3*_	0.14	0.11	1.55	.123	-0.07	0.35
X*W *R*^*2*^ change = .009, *F*(1, 271) = 2.39, *p* = .123							
Model 2. Health-related quality of life change							
*R*^*2*^ = 0.054, *MSE* = 394.70, *F*(3, 271) = 5.13, *p* = .002							
Constant	*i*_*y*_	17.23	1.58	10.52	.000	14.11	20.34
AWL 24 m (X)	*b*_*1*_	0.48	0.13	3.62	.000	0.22	0.73
Chronic pain (W)	*b*_*2*_	-1.34	2.45	-0.56	.578	-6.42	3.28
Interaction	*b*_*3*_	-0.25	0.21	-1.24	.216	-0.68	0.16
X*W *R*^*2*^ = .005, *F*(1, 271) = 1.54, *p* = .216							
Model 3. Self-esteem change							
*R*^*2*^ = 0.036, *MSE* = 18.79, *F*(3, 265) = 3.32, *p* = .021							
Constant	*i*_*y*_	2.48	0.38	6.86	.000	1.75	3.22
AWL 24 m (X)	*b*_*1*_	0.08	0.03	2.78	.006	0.02	0.15
Chronic pain (W)	*b*_*2*_	-0.36	0.52	-0.68	.499	-1.39	0.65
Interaction	*b*_*3*_	-0.03	0.04	-0.70	.482	-0.12	0.05
X*W *R*^*2*^ = .002, *F*(1, 265) = 0.495, *p* = .482							
Model 4. Self-efficacy exercise change							
*R*^*2*^ = 0.027, *MSE* = 37.23, *F*(3, 271) = 2.54, *p* = .057							
Constant	*i*_*y*_	2.72	0.50	5.41	.000	1.72	3.68
AWL 24 m (X)	*b*_*1*_	0.10	0.04	2.49	.013	0.03	0.18
Chronic pain (W)	*b*_*2*_	-0.52	0.73	-0.70	.485	-1.94	0.91
Interaction	*b*_*3*_	-0.05	0.07	-0.82	.415	-0.19	0.08
X*W *R*^*2*^ = .002, *F*(1, 271) = 0.67, *p* = .415							
Model 5. Physical activity change							
*R*^*2*^ = 0.003, *MSE* = 29011.29, *F*(3, 271) = 0.30, *p* = .827							
Constant	*i*_*y*_	40.22	14.15	2.86	.005	12.12	67.51
AWL 24 m (X)	*b*_*1*_	-0.67	1.11	-0.59	.554	-2.85	1.53
Chronic pain (W)	*b*_*2*_	6.61	20.73	0.32	.750	-33.25	47.78
Interaction	*b*_*3*_	-0.25	1.81	-0.14	.887	-3.76	3.31
X*W *R*^*2*^ = .0001, *F*(1, 271) = 0.02, *p* = .887							
Model 6. Self-efficacy eating change							
*R*^*2*^ = 0.036, *MSE* = 357.67, *F*(3, 271) = 3.34, *p* = .020							
Constant	*i*_*y*_	9.75	1.62	6.25	.000	6.65	12.97
AWL 24 m (X)	*b*_*1*_	0.38	0.13	3.03	.003	0.12	0.64
Chronic pain (W)	*b*_*2*_	-1.34	2.30	-0.58	.560	-5.97	3.11
Interaction	*b*_*3*_	-0.49	0.19	-2.57	.011	-0.88	-0.12
X*W *R*^*2*^ = .024, *F*(1, 271) = 6.62, *p* = .011							
Model 7. Food craving change							
*R*^*2*^ = 0.041, *MSE* = 371.23, *F*(3, 268) = 3.82, *p* = .011							
Constant	*i*_*y*_	-11.07	1.63	-6.92	.000	-14.33	-7.91
AWL 24 m (X)	*b*_*1*_	-0.39	0.14	-3.03	.003	-0.66	-0.13
Chronic pain (W)	*b*_*2*_	-2.17	2.32	-0.92	.357	-6.72	2.40
Interaction	*b*_*3*_	0.24	0.21	1.21	.228	-0.17	0.65
X*W *R*^*2*^ = .005, *F*(1, 268) = 1.46, *p* = .228							

*Coeff*, coefficients; *SE*, standard error; *LLCI*, lower limit of 95% confidence interval; *ULCI*, upper limit of 95% confidence interval. Bias-corrected bootstrap

*n* = 275 for all models except Model 3 (*n* = 269) and 7 (*n* = 272)

The first model estimated the change in depression from pre-operation to 24 months post-operation. The main effect of %AWL at 24 months on depression change (*b* = -0.02, *p* = 0.74), and the main effect of chronic pain on depression change (*b* = 0.00) were insignificant. Similarly, the interaction effect was also insignificant (*b* = 0.14, *p* = 0.12) and only explained 0.9% of the variance in depression, *F*(1, 271) = 2.39, *p* = 0.12. Thus, a moderation effect was not present.

No moderation effects of chronic pain on change in health-related quality of life, self-esteem, and self-efficacy to exercise could be concluded. Only the main effect of %AWL was significant in these models, suggesting that greater weight loss was associated with greater improvement in the outcome variables. None of the other effects were significant in these models. Similarly, only the main effect of %AWL on change in food cravings was also significant. Specifically, greater weight loss was associated with greater amelioration in food cravings (*b* = -0.39, *p* = 0.003). Furthermore, no significant effects were found between %AWL, physical activity and pain syndromes.

**Fig. 1:**
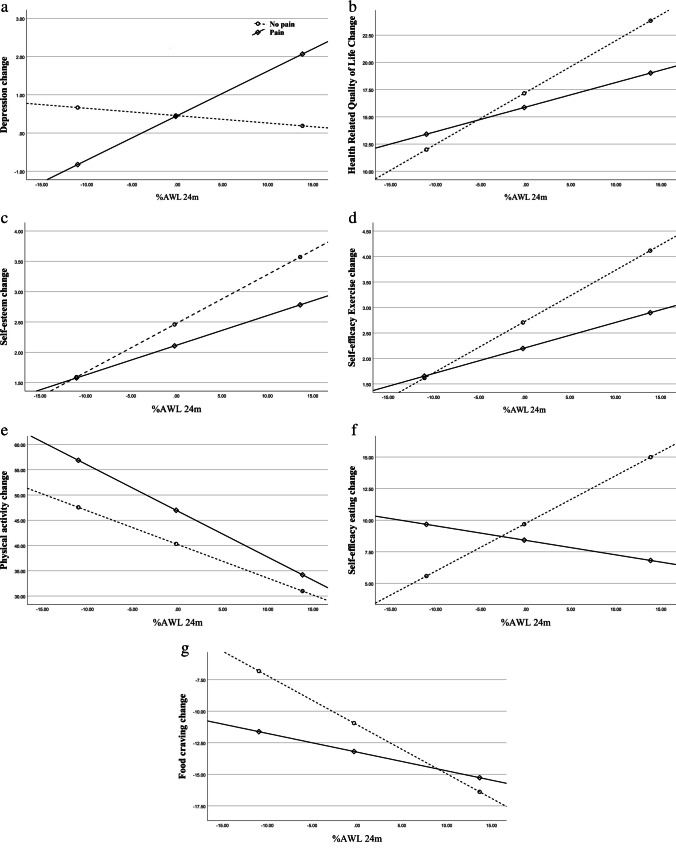
Chronic pain as a moderator between weight loss 24 months after surgery and change in psychological wellbeing: **a**) depression, **b**) HR-Quality of Life, and **c**) Self-esteem. Solid line = chronic pain group, dashed line = no chronic pain group. Chronic pain as a moderator between weight loss 24 months after surgery and change in psychological wellbeing: **d**) Self-efficacy to exercise, **e**) Physical activity, **f**) Self-efficacy to control eating behaviours, and **g**) Food cravings

In the model with self-efficacy to control eating behaviours, there was a significant main effect of %AWL, *b* = 0.38, CI [0.12, 0.64], *p* < 0.01, and nonsignificant main effect of chronic pain on self-efficacy to control eating behaviours, *b* = -1.34, CI [-5.97, 3.11], *p* = 0.56. However, there was a significant interaction found by presence of pain syndromes on %AWL and self-efficacy to control eating, *b* = -0.49, CI [-0.88, -0.12], which explained 2.4% (*F*(1, 271) = 6.62, *p* = 0.011) of the variance in change in self-efficacy to control eating behaviours beyond the main effects. The effect of weight loss was significantly different from zero for patients without pain syndromes *b* = 0.38, CI [0.13, 0.63], *p* = 0.003, while the effect was not significant for patients with pain syndromes, *b* = -0.12, CI [-0.40, 0.17], *p* = 0.43 (Fig. 1). The simple slope indicated that for patients without pain syndromes, higher weight loss was associated with greater improvement in self-efficacy to control eating.

## Discussion

This study set out to examine the impact of chronic pain on psychological functioning and weight loss among patients with obesity undergoing bariatric metabolic surgery. In our sample, 46% of patients reported one or more chronic pain conditions. The most prevalent chronic pain categories were chronic musculoskeletal and chronic primary pain, with the most common specific conditions being chronic lower back pain or pain due to osteoarticular change (38.8%), followed by migraine (9.1%), fibromyalgia (8.7%), and IBS (6.9%). Contrary to our hypotheses, the moderation analyses showed that pain syndromes did not moderate the associations between weight loss and changes in most psychological outcomes. In other words, the 24-month postoperative weight loss was associated with similar improvement in psychological outcomes between patients with and without preoperative pain syndromes. However, pain syndromes moderated the relationship between weight loss and change in self-efficacy to control eating behaviours 24 months after bariatric metabolic surgery. Specifically, this improvement in self-efficacy to control eating behaviours associated with weight loss was only observed among patients without preoperative pain syndromes. Another noteworthy finding was that greater weight loss was associated with greater improvement in health-related quality of life, self-esteem, self-efficacy to exercise, and greater amelioration in food cravings, in patients without pain as well as patients with pain syndromes. These results are in line with those of previous studies demonstrating the positive effect of bariatric metabolic surgery and weight loss on psychological health [24, 25, 47]. Interestingly, however, change in physical activity was not related to weight loss.

Our study is the first to investigate chronic pain syndromes and their effect on various psychological outcomes after bariatric metabolic surgery. Earlier studies have mainly focused on changes in severity, frequency and location of pain and its potential impact on weight loss, neglecting the impact pain may have on psychological health. Interestingly, only a few differences were found in preoperative psychological characteristics between patients with and without pain syndromes. Patients with pain had poorer pre-operative health-related quality of life and more depressive symptoms, but similar scores on self-efficacy, physical activity levels, food cravings, personality characteristics (i.e., neuroticism and conscientiousness), attachment style and self-esteem. This result suggests that the presence of pain syndromes may reflect as poorer preoperative psychological health, while patients’ personality characteristics and health behaviours remain largely unaffected.

As expected, weight loss did not differ between patients with or without a pain syndrome. This is in contrast with a recent study, which found greater pain to be related to poorer weight loss and greater weight regain after bariatric metabolic surgery [48]. Furthermore, patients’ subjective pain scores improved during the first 36 months after bariatric metabolic surgery, and slowly deteriorated thereafter [48]. However, the aforementioned study measured pain in magnitude, which could explain the difference between our findings. The impact of pain on weight loss may depend on the severity rather than the presence of pain. It is also possible that the effects of pain severity on weight outcomes become more evident in the longer term, after the strong surgical effects on weight loss wear off [49] and weight maintenance depends more on the patient themselves [49].

We did not find a relationship between pain syndromes and health behaviours (i.e., physical activity and food cravings) during the first 24 months after surgery, although prior studies have shown that chronic pain may hinder physical activity, performance of daily activities and self-efficacy to exercise [14, 17, 18, 50, 51]. Furthermore, patients with chronic pain and medical problems associated with obesity tend to eat energy-dense and palatable foods to soothe their pain, which may lead to further weight gain among patients with obesity [19, 22]. This may be relevant for long-term weight regain (2–7 years after the operation) and should be considered during treatment. Future research should focus on the mechanisms underpinning pain and postoperative health behaviours.

### Strengths and Limitations

This study is limited by the absence of pain scales measuring magnitude and location of pain. We did not measure pain at the postoperative visits, thus we could not assess whether pain syndromes ameliorated after bariatric metabolic surgery. In order to obtain precise information regarding changes in pain after weight loss surgery, future studies should include these measures and actively enquire whether pain syndromes of patients with obesity ameliorate post-surgery. Since the study is limited to patients undergoing bariatric metabolic surgery, it is not possible to generalize these results to all patients with obesity or overweight. One potential limitation of our data may arise from selection bias introduced by two factors: firstly, the inclusion of only those patients with complete data from all assessments in the analyses, and secondly, the inclusion of patients participating in the study who consented to additional testing and biopsy procedures during their surgeries. Nevertheless, the clinical attributes and prevalence of pain syndromes in this group closely resemble those of all patients treated at our medical facility, with the sole distinction being a mean age that is five years higher. Lastly, our study was limited by the relatively small sample size. We recommend further studies to include a longer follow-up period to investigate how chronic pain syndromes may develop over time and what their impact is on psychological functioning. Further work exploring the impact of the separate pain conditions on health outcomes, is needed to fully understand the implications of pain conditions on the progression of psychological and physical health among patients undergoing bariatric metabolic surgery. Nonetheless, the authors believe this study also has several strengths, including longitudinal assessment, and measuring various psychological characteristics and outcomes over time. In spite of its limitations, this study adds to our understanding of the positive impact weight loss has on postoperative improvements in psychological wellbeing and health behaviours (health-related quality of life, self-efficacy to control eating behaviours, self-esteem, and food cravings) and how pain syndromes may pose additional challenges to patients’ psychological wellbeing.

## Conclusion

The results of this study suggest that patients with obesity undergoing bariatric metabolic surgery with pain syndromes have poorer preoperative psychological wellbeing (i.e., depression and health-related quality of life) than those without pain syndromes, whereas patients without pain may have less difficulties to live healthily after the operation as they experienced greater improvement in self-efficacy to control eating behaviour the more weight they lost. However, greater weight loss was related to improving psychological functioning during the first 24 months after surgery for all patients in the study. Continued advances in our understanding of the relationship between pain and psychological functioning (e.g., self-efficacy, self-esteem, quality of life), both short- and long term after the surgery, may help to optimize follow-up and intervention strategies for patients after bariatric metabolic surgery.

## Data Availability

Deidentified study data will be shared upon reasonable request by contacting the corresponding author. The full study protocol is published and available to the public [31].

## Abbreviation

Δ: Delta.
